# Leucine-Rich Repeat Kinase 2 Controls Inflammatory Cytokines Production through NF-κB Phosphorylation and Antigen Presentation in Bone Marrow-Derived Dendritic Cells

**DOI:** 10.3390/ijms21051890

**Published:** 2020-03-10

**Authors:** Makoto Kubo, Ryuichi Nagashima, Mitsue Kurihara, Fumitaka Kawakami, Tatsunori Maekawa, Koji Eshima, Etsuro Ohta, Hirotomo Kato, Fumiya Obata

**Affiliations:** 1 Division of Immunology II, Kitasato University School of Allied Health Sciences, 1-15-1 Kitasato, Minami-ku, Sagamihara, Kanagawa 252-0373, Japan; 2 Division of Clinical Immunology, Graduate School of Medical Sciences, Kitasato University, 1-15-1 Kitasato, Minami-ku, Sagamihara, Kanagawa 252-0373, Japan; 3 Research Facility of Regenerative Medicine and Cell Design, Kitasato University School of Allied Health Sciences, 1-15-1 Kitasato, Minami-ku, Sagamihara, Kanagawa 252-0373, Japan; 4 Laboratory of Molecular Signal Biology, Graduate School of Medical Sciences, Kitasato University, 1-15-1 Kitasato, Minami-ku, Sagamihara, Kanagawa 252-0373, Japan; 5 Department of Immunology, Kitasato University School of Medicine, 1-15-1 Kitasato, Minami-ku, Sagamihara, Kanagawa 252-0373, Japan; 6 Division of Medical Zoology, Department of Infection and Immunity, School of Medicine, Jichi Medical University, 3311-1 Yakushiji, Shimotsuke, Tochigi 329-0498, Japan; 7 Kitasato Junior Colledge of Health and Hygienic Sciences, 500 Kurotsuchi-Shinden, Minami-Uonuma, Niigata 949-7241, Japan

**Keywords:** leucine-rich repeat kinase 2, Parkinson’s disease, bone marrow-derived dendritic cells, *Leishmania*

## Abstract

Leucine-rich repeat kinase 2 (LRRK2) is the causal molecule of familial Parkinson’s disease. Although the characteristics of LRRK2 have gradually been revealed, its true physiological functions remain unknown. LRRK2 is highly expressed in immune cells such as B2 cells and macrophages, suggesting that it plays important roles in the immune system. In the present study, we investigate the roles of LRRK2 in the immune functions of dendritic cells (DCs). Bone marrow-derived DCs from both C57BL/6 wild-type (WT) and LRRK2 knockout (KO) mice were induced by culture with granulocyte/macrophage-colony stimulating factor (GM/CSF) in vitro. We observed the differentiation of DCs, the phosphorylation of the transcriptional factors NF-κB, Erk1/2, and p-38 after lipopolysaccharide (LPS) stimulation and antigen-presenting ability by flow cytometry. We also analyzed the production of inflammatory cytokines by ELISA. During the observation period, there was no difference in DC differentiation between WT and LRRK2-KO mice. After LPS stimulation, phosphorylation of NF-κB was significantly increased in DCs from the KO mice. Large amounts of inflammatory cytokines were produced by DCs from KO mice after both stimulation with LPS and infection with *Leishmania*. CD4^+^ T-cells isolated from antigen-immunized mice proliferated to a significantly greater degree upon coculture with antigen-stimulated DCs from KO mice than upon coculture with DCs from WT mice. These results suggest that LRRK2 may play important roles in signal transduction and antigen presentation by DCs.

## 1. Introduction

Leucine-rich repeat kinase 2 (LRRK2) is the causative molecule of autosomal-dominant familial Parkinson’s disease (PD) [1,2,3,4]. It has an approximate molecular weight of 280 kDa and contains multiple domain structures, including the leucine-rich repeat (LRR), ras of complex (ROC), C-terminal of ROC (COR), kinase, and WD40 domains [3,4,5]. Although it has been reported that the kinase activity of LRRK2 has some association with the pathogenesis of PD, inflammatory bowel disease (IBD), and some bacterial infections [6,7], its true function remains unclear.

Many studies of human and animal brains have indicated that LRRK2 is present in various regions such as the cerebellum, cortex, putamen, and substantia nigra [3,4,8,9,10,11,12]. Moreover, LRRK2 shows much higher expression in lung, spleen, kidney, and testis than in other organs [12,13,14,15,16]. We and others have previously reported that B cells and macrophages in mouse spleen also express LRRK2 protein, and that expression of LRRK2 mRNA in B2 cells, one of the B cell subsets, is much higher than that in B1 cells [16,17,18,19,20]. These findings suggest that LRRK2 has important roles in not only the nervous system but also the immune system. In fact, we have also shown that LRRK2-knockout (KO) mice have a higher proportion and number of peritoneal B1 cells and splenic B2 cells than wild-type (WT) mice, and that LRRK2 regulates extracellular signal-regulated kinase (Erk)1/2 phosphorylation in splenic B2 cells after anti-IgM antibody stimulation. We have also demonstrated that LRRK2 KO mice have a higher serum IgA level and enhanced T-cell-independent antigen responses [21].

LRRK2 expression is also evident in myeloid cells, such as macrophages and dendritic cells (DCs) [22], which play important roles in phagocytosis and an antigen presentation to T-cells. Both of these functions are intimately linked to autophagy, and LRRK2 expression in DCs has also been shown to have an association with autophagy in a mouse model [23]. The perturbation of autophagy function is known to be related to IBD, PD, and intracellular infection with pathogens such as *Mycobacterium tuberculosis* and *M*. *leprae* [6,7]. LRRK2 is known to act as a regulator of autophagy in immune cells through increased translocation to the autophagosome membrane after LPS stimulation [24,25,26]. Recently, it has been demonstrated that LRRK2 is also recruited to lysosomes [27]. Therefore, LRRK2 in DCs may play important roles in both phagocytosis and antigen-presentation via its autophagic function.

In the present study, we investigate whether LRRK2 expression in DCs influences the stage of differentiation, signal transduction after stimulation, and antigen presentation. We show that mice with LRRK2 knockout (KO) have higher production of inflammatory cytokines after stimulation with lipopolysaccharide (LPS) and infection with parasites than WT mice and that LRRK2 negatively regulates the phosphorylation of nuclear factor-kappa B (NF-κB) in DCs after LPS stimulation. We also demonstrate that LRRK2-KO mice show a higher degree of antigen presentation to CD4^+^ T-cells isolated from both antigen-immunized WT mice and KO mice. 

## 2. Results

### 2.1. Differentiation of BMDCs

**Figure 1:**
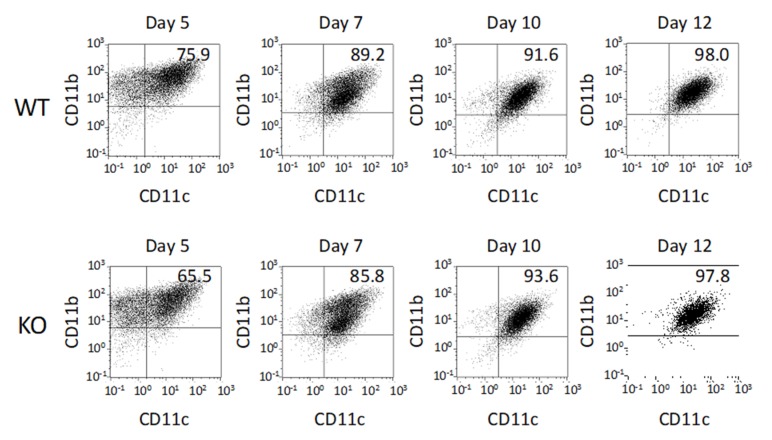
Differentiation of bone marrow derived dendritic cells (BMDCs). BMDCs induced from WT and KO mice were characterized by flow-cytometric analysis after staining with immunofluorescent antibodies against CD11b and CD11c, as described in Materials and Methods. The upper panels show the course of differentiation of BMDCs induced from WT mice and the lower panels that of BMDCs from LRRK2–KO mice. Data are representative of three independent experiments each involving six mice (WT *n* = 3, KO *n* = 3).

To confirm the differentiation of bone marrow derived dendritic cells (BMDCs) from both WT and KO mice, we carried out flow cytometric analysis. The proportion of CD11b^+^CD11c^+^ cells gradually increased during incubation. After 10 days of incubation, more than 90% of these cells had differentiated to CD11b^+^CD11c^+^ BMDCs. There were no significant differences in CD11b^+^CD11c^+^ BMDC differentiation between WT and KO mice (Figure 1). After BMDC differentiation, we investigated that LRRK2 expression in those from both WT and KO mice. LRRK2 expression in WT BMDCs was only confirmed by Western blotting (Figure S1).

### 2.2. Cytokine Production and Signal Analysis of BMDCs

**Figure 2:**
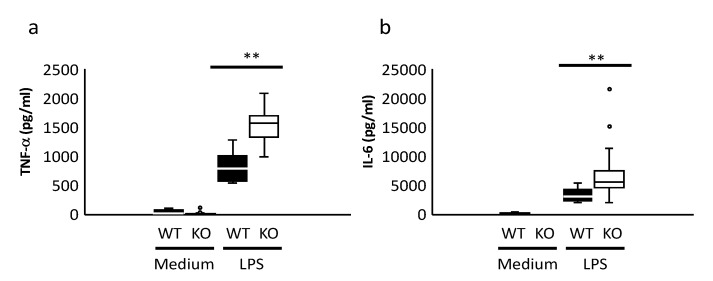
Production of inflammatory cytokines from lipopolysaccharide (LPS)-stimulated BMDCs. BMDCs induced from wild-type (WT) and knockout (KO) mice were stimulated by LPS. After 24 h, the culture supernatants were collected, and the concentrations of TNF-α and IL-6 were determined by ELISA, as described in Materials and Methods. (**a**) TNF-α levels in the culture supernatants of BMDCs from WT (black bar) and KO (white bar) mice. (**b**) IL-6 levels in the culture supernatants of BMDCs from WT (black bar) and KO (white bar) BMDCs. Data in both (**a**) and (**b**) are collected the data of three independent experiments each involving six mice (WT *n* = 3, KO *n* = 3) and analyzed by one-way ANOVA with Bonferroni post hoc test. ** *p* < 0.01.

As shown in Figure 1, there was no significant difference in BMDC differentiation between WT and KO mice. To investigate the function of differentiated BMDCs, we next examined whether there were any differences between those from in terms of tumor necrosis factor-α (TNF-α) and interleukin-6 (IL-6) secreted into culture supernatants following LPS stimulation. After 24 h of incubation, BMDCs from KO mice showed significantly higher TNF-α and IL-6 production than those from WT mice (Figure 2a,b), suggesting that LRRK2 might regulate toll-like receptor 4 (TLR4) signaling.

**Figure 3:**
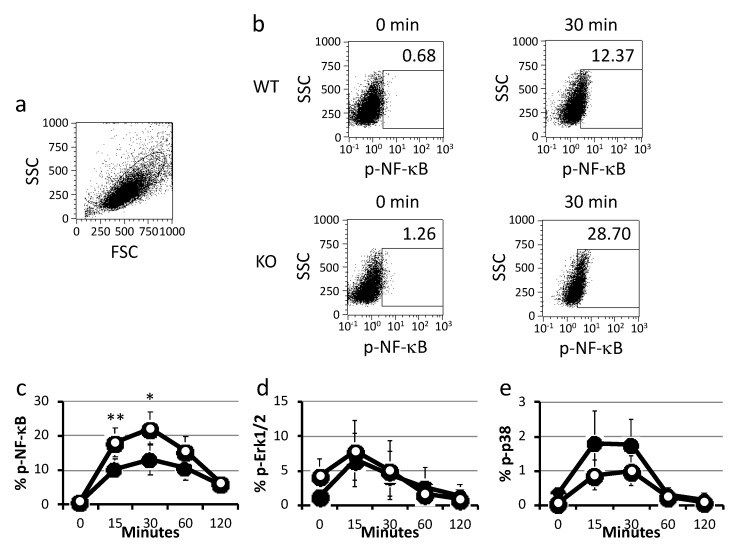
Phosphorylation of transcriptional factors in LPS-stimulated BMDCs. (**a**) Gating of BMDCs, which were induced from WT (*n* = 9) and KO (*n* = 9) mice. (**b**) Expression of phosphorylated NF-κB (Ser536) in LPS-stimulated BMDCs from WT and KO mice as revealed by flow-cytometry (see Materials and Methods). Data are representative of three independent experiments each involving six mice (WT *n* = 3, KO *n* = 3) (**c**) Proportions of phosphorylated NF-κB (Ser536) in BMDCs induced from WT (black circles) and KO (white circles) mice after LPS stimulation. (**d**) Data shown are the proportions of phosphorylated Erk1/2 (Thr202/Tyr204) in BMDCs from WT (black circles) and KO (white circles) mice after LPS stimulation. (**e**) Proportions of phosphorylated p-38 (pT180/pY182) in BMDCs from WT (black circles) and KO (white circles) mice after LPS stimulation. Data are collected the data of two independent experiments each involving six mice (WT *n* = 3, KO *n* =3) analyzed by two-way ANOVA with Bonferroni post hoc test. * *p* < 0.05, ** *p* < 0.01.

TLR4 signaling triggered by LPS stimulation can activate NF-κB and p-38 signaling. Moreover, we have previously reported that the expression of phosphorylated Erk1/2 is much higher in B2 cells after B cell receptor signaling. To study the efficacy of BMDCs activation by LPS stimulation, we next examined whether phosphorylation of NF-κB (Ser536), p-38 (pT180/pY182), and Erk1/2 (pT202/pY204) was influenced by LPS stimulation in BMDCs from WT and KO mice. After LPS stimulation, BMDCs were collected and stained for intracellular phosphorylated NF-κB (Ser536), p-38 (pT180/pY182), and Erk1/2 (pT202/pY204) using anti-phospho antibodies. Expression of phosphorylated NF-κB (Ser536) in BMDCs from KO mice was significantly higher than that in BMDCs from WT mice at 15 and 30 min after inoculation (Figure 3a–c), whereas the expression of phosphorylated p-38 (pT180/pY182) and Erk1/2 (pT202/pY204) showed no significant differences between the two (Figure 3d,e).

### 2.3. Assessment of BMDC Antigen-Presenting Ability

**Figure 4:**
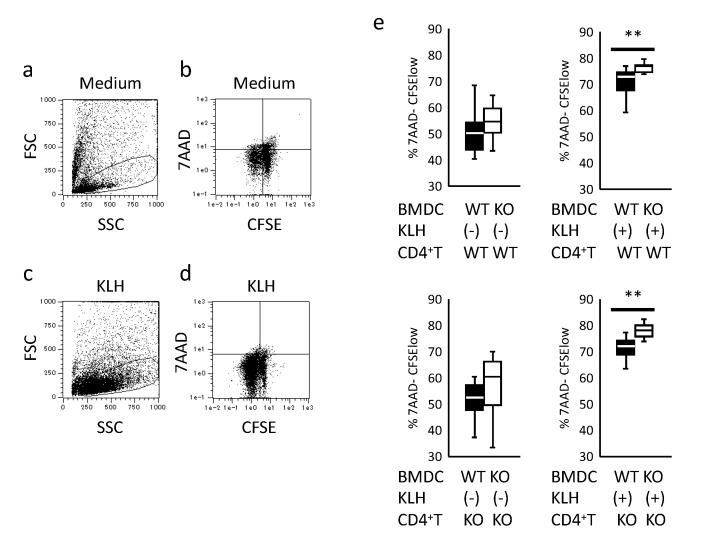
Antigen presentation by BMDC and antigen-immunized CD4^+^ T-cells proliferation. CD4^+^ T-cells were isolated from WT and KO mice immunized with keyhole-limpet hemocyanin (KLH). CD4^+^ T-cells were co-cultured with KLH-stimulated BMDCs, as described in Materials and Methods. (**a**) Gating of CD4^+^ T-cells cultured with unstimulated KO BMDCs. (**b**) Proliferation of CD4^+^ T-cells cultured with unstimulated KO BMDCs. (**c**) Gating of CD4^+^ T-cells cultured with KLH-stimulated KO BMDCs. (**d**) Proliferation of CD4^+^ T-cells cultured with KLH-stimulated KO BMDCs. (**e**) Proliferation of CD4^+^ T-cells isolated from KLH-immunized WT and KO mice and co-cultured with unstimulated and KLH-stimulated BMDCs from WT and KO mice. Data are collected the data of two independent experiments each involving eight mice (WT *n* = 4, KO *n* = 4) analyzed by Student’s *t* test. ** *p* < 0.01.

Antigen presentation to T-cells is one of the main functions of DCs. To investigate the antigen-presenting ability of BMDCs, we immunized WT and KO mice with keyhole-limpet hemocyanin (KLH) mixed with Freund’s complete adjuvant. At 7–10 days after immunization, popliteal and inguinal lymph nodes of immunized WT and KO mice were collected as KLH-primed CD4^+^ T-cell sources. Then, isolated CD4^+^ T-cells from immunized WT and KO mice were stained with 5(6)-carboxyfluorescein diacetate succinimidyl ester (CFSE). CFSE-stained CD4^+^ T-cells were co-cultured with KLH-stimulated BMDCs from WT and KO mice. After 3 days of incubation, the co-cultured cells were collected and analyzed by flow cytometry. The proliferation of WT and KO CD4^+^ T-cells co-cultured with unstimulated BMDCs from KO mice was not significantly altered (Figure 4a,b,e, upper and lower left panels). In contrast, the proliferation of WT and KO CD4^+^ T-cells co-cultured with KLH-stimulated BMDCs from KO mice was significantly increased relative to that of BMDCs from WT mice (Figure 4c–e, upper and lower right panels). To further investigate the higher proliferation of CD4^+^ T-cells induced by BMDCs from KO mice, we determined the expression of CD86 and major histocompatibility complex class II (MHC-II) on the BMDCs. These molecules expressed on antigen-presenting cells play important roles in CD4^+^ T-cell activation. Expression of CD86 in BMDCs from KO mice tended to be higher expression than that in BMDCs from WT mice (Figure S2), whereas MHC-II expression did not differ between WT and KO BMDCs (data not shown).

### 2.4. Influence of Infection on LRRK2–KO Mice

**Figure 5:**
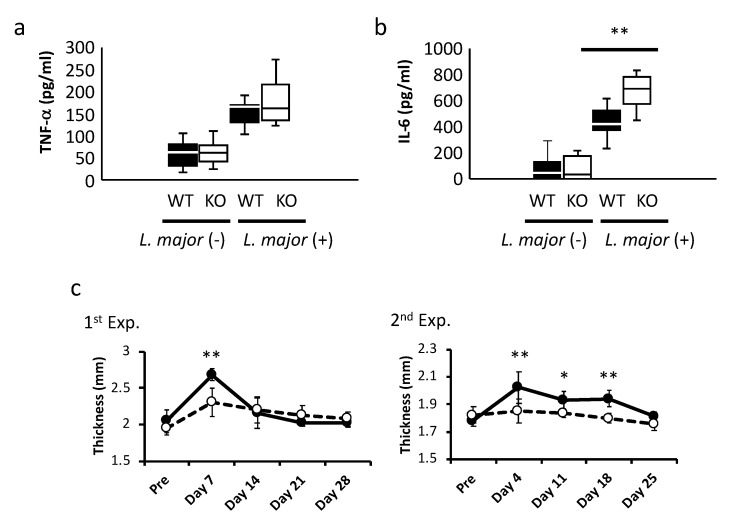
*Leishmania* (*Leishmania*) *major* infection in vitro and in vivo. BMDCs from WT and KO mice were infected with *L.* (*L.*) *major*. After 3 days of incubation, the culture supernatants were collected, and the concentration of IL-6 were measured by ELISA, as described in Materials and Methods. (**a**) TNF-α levels in the culture supernatants of WT (black bar) and KO (white bar) BMDCs. (**b**) IL-6 levels in the culture supernatants of WT (black bar) and KO (white bar) BMDCs. Data in both (**a**) and (**b**) are collected the data of three independent experiments each involving six mice (WT *n* = 3, KO *n* = 3) analyzed by one-way ANOVA with Bonferroni post hoc test. ** *p* < 0.01. WT and KO mice were infected with *L.* (*L.*) *major* (1 × 10^6^ metacyclics) in footpads. (**c**) Thickness of *L.* (*L.*) *major-*infected footpads in WT (black circles) and KO (white circles) mice. The left panel shows the first experiment with 12 mice (WT *n* = 5, KO *n* = 7) and the right panel shows the second experiment with 13 mice (WT *n* = 6, KO *n* = 7) analyzed by two-way ANOVA with Bonferroni post hoc test. * *p* < 0.05, ** *p* < 0.01.

As BMDCs from KO mice showed higher production of inflammatory cytokines and greater antigen-presenting ability than those from WT mice, it is possible that the two types of mice may show some differences in their response to infection. A genome-wide association study has shown that LRRK2 is associated with inflammatory bowel disease (IBD) and *Mycobacterium leprae* infection. *M. leprae* can infect phagocytic cells as an intracellular pathogen. *Leishmania* (*Leishmania*) *major* is a protozoan, but can also be an intracellular pathogen if it is phagocytized. To investigate whether LRRK2 can mediate infection with intracellular pathogens, we co-cultured BMDCs with *L.* (*L.*) *major* for 3 days in vitro, then measured the levels of inflammatory cytokines secreted into the culture supernatants using ELISA. The concentration of IL-6 in supernatants of BMDCs from LRRK2–KO mice was significantly higher than that in supernatants of BMDCs from WT mice, while the concentration of TNF-α in supernatants of BMDCs from LRRK2–KO mice tended to be high (Figure 5a,b). To further study in vivo infection, we also inoculated WT and KO mice with *L.* (*L.*) *major*, then periodically measured the thickness of their footpads. Unexpectedly, the footpad thickness of WT mice footpads was greater than that of KO mice (Figure 5c). Using a multi-analyte flow assay kit, we also measured the levels of Th1/Th2 cytokines in serum samples taken from WT and KO mice 4 days after infection with *L.* (*L.*) *major*. However, no inflammatory cytokines were detected in serum (data not shown).

## 3. Discussion

This study is performed to determine whether LRRK2, a complex kinase that had been originally been identified as the causative molecule of familial and sporadic Parkinson’s disease, plays important roles in cytokine production and antigen-presenting ability in BMDCs. In LRRK2–KO mice, we demonstrated that expression of the inflammatory cytokines TNF-α and IL-6 in BMDCs was significantly higher than in WT mice after LPS stimulation. We found that the degree of NF-κB phosphorylation (Ser536) was higher in KO mice than in WT mice, and that BMDCs from KO mice showed significantly higher ability to present antigen to KLH-primed CD4^+^ T-cells than those from WT mice. We also observed that BMDCs from KO mice inoculated in vitro with *L.* (*L.*) *major* produced higher amount of IL-6 in the culture supernatants, whereas WT mice inoculated with *L.* (*L.*) *major* in vivo developed significantly thicker footpads than KO mice.

Dendritic cells are specialized cells with potent antigen-presenting ability. Gardet et al. reported that LRRK2 is expressed by dendritic cells in the lamina propria [22]. In this study, we also confirm that LRRK2 was expressed by BMDCs from WT mice but not by those from KO mice. We also observed no differences in BMDC differentiation between WT and LRRK2-KO mice, suggesting that LRRK2 might not be associated with the regulation of BMDC differentiation. Liu et al. previously reported that bone marrow-derived macrophages (BMDMs) from LRRK2-deficient mice show enhanced production of IL-6 and IL-12 p40 after stimulation with zymosan [28]. Here, we also observe that BMDCs from LRRK2-KO mice showed enhanced production of TNF-α and IL-6 after LPS stimulation. Liu et al. have shown that NFAT activation is associated with the higher production of IL-6 and IL-12 p40 by BMDMs from LRRK2-deficient mice, whereas we found that the NF-κB phosphorylation (Ser536) in BMDCs from KO mice was higher than that in BMDCs from WT mice. Although the reason for these differences between BMDCs and BMDMs in inflammatory production of cytokines is unclear, expression to various types of stimulators may play a role. We have previously reported that Erk1/2 phosphorylation (Thr202/Tyr204) of splenic B2 cells was higher in KO mice than in WT mice after α-IgM stimulation [21]. In the present study, we found that there was no significant difference in Erk1/2 phosphorylation of BMDCs between WT and KO mice. This may have been due to differences between TLR4 and B cell receptor (BCR) signaling. BCR signaling involves at least two pathways: Akt1 activation via phosphatidylinositol 3-kinase (PI3K), and Erk1/2 activation through phospholipase C-γ 2 (PLCγ2) [29]. TLR4 signaling also involves at least two pathways: NF-κB and IRF3 [30]. NF-κB is activated by Akt1 via PI3K downstream of MyD88, and IRF3 is activated by TRIF. We have reported previously that Akt1 (Ser473) is directly phosphorylated by LRRK2 [31]. In the present study, we found that NF-κB phosphorylation in BMDCs from KO mice was significantly higher than that in BMDCs from WT mice after LPS stimulation, suggesting that LRRK2 may directly or indirectly operate as a negative regulator of NF-κB activation. However, we did not detect the production of interferon-β (IFN-β) after LPS stimulation (data not shown), and therefore LRRK2 may not be involved in the IRF3 pathway.

LRRK2 in DCs has also been shown to be associated with autophagy in a mouse model. Therefore, we investigated whether LRRK2 expression in BMDCs would affect antigen presentation to CD4^+^ T-cells isolated from KLH-immunized WT and KO mice. We found that BMDCs from KO mice stimulated a significant proliferation of CD4^+^ T-cells isolated from KLH-immunized WT and KO mice. There was no significant difference in CD4^+^ T-cell proliferation between WT and KO mice after KLH immunization. This suggests that the difference of CD4^+^ proliferation was dependent on the antigen presentation ability of BMDCs and their expression of costimulatory molecules. We also found that the expression of CD86, but not MHC-II, by BMDCs from KO mice tended to be high after LPS stimulation (Figure S2 and data not shown). LRRK2 may, therefore, affect CD86 expression after physiological stimulation. Takagawa et al. recently reported that LRRK2 overexpression in BMDCs inhibited autophagy through the inactivation of Declin-1 [23]. Our present result suggests that LRRK2 may also negatively regulate the efficacy of antigen presentation via the autophagic pathway.

Alpha-synuclein (α-syn) and LRRK2 are highly associated with PD. α-Syn accumulation in Lewy bodies has been well documented in the brain of PD patients. Previous reports have demonstrated that nitric oxide produced by oxidative stress generates nitration of the tyrosine residues of α-syn. Nitrated α-syn extracellularly released is taken up by microglia, which is one of the immune cells in the central nervous system, and elevates the expression of TNF-α, TNF receptor 1, and nitric oxide synthase 2 following nuclear localization of NF-κB. This nitrated α-syn activation of microglia can promote innate and adaptive immune responses [32,33]. Moreover, the association between leprosy and LRRK2 has been reported by a genome-wide association study (GWAS). During leprosy and after bacteria clearance, 30–50% of cases alter towards a pro-inflammatory response, termed type-1 reactions (T1R). LRRK2 mutation is associated with an increase of T1R [34]. The association of infections and neurodegenerative diseases have been reported in experimental models and epidemiologic studies [35]. The increase of α-syn expression has been induced by the West Nile virus infection [36]. Hepatitis C virus infection has demonstrated the association of the PD risk in cohort studies [37]. LRRK2 is highly expressed in, not only DC, but also microglia. LRRK2 mutation and the alteration of expression level induced by infection and peripheral inflammation may affect the activation and function of DC and microglia in neurodegenerative disorders, such as PD. Accordingly, we will need to investigate inflammatory activities of DC and microglia induced by α-syn in LRRK2–KO mice.

*L.* (*L.*) *major* is an intracellular pathogen of phagocytic cells. Here, we also confirmed that the production of inflammatory cytokines by BMDCs from KO mice was significantly higher than that of BMDCs from WT mice after in vitro infection with *L.* (*L.*) *major*, suggesting that *L.* (*L.*) *major* pathogenesis in KO mice was aggravated by high production of inflammatory cytokines. In vivo, however, we observed that footpads of WT mice infected with *L.* (*L.*) *major* were thicker than those of similarly infected KO mice. In comparison with WT mice, *L.* (*L.*) *major* in KO mice might be eliminated earlier as a result of high inflammatory cytokine production. No TNF-α and IL-6 were detected in serum from both WT and KO mice at 4 days after *L.* (*L.*) *major* infection. Further analysis is needed to clarify the difference between in vitro and in vivo infection in this setting. Other infectious parasite diseases have been reported in association with neurodegenerative disorders, such as PD. *Toxoplasma gondii* (*T. gondii*) is an intracellular protozoa parasite and one of the worldwide health problems. Miman et al. have demonstrated the seropositive rates of anti- *T. gondii* IgG antibodies in PD patients are higher than in control groups [38]. Li et al. have reported *T. gondii* infection induces microglia activation via a chemokine (CX3CL1) expression and complement proteins (C1q and C3) upregulation [39]. Moreover, *Trypanosoma cruzi* (*T. cruzi*) is also an intracellular protozoa parasite and the cause of Chagas’ disease. A parasite-derived neurotropic factor (PDNF) produced by *T. cruzi* can directly activate tyrosine hydroxylase, an enzyme involving in dopamine synthesis [40]. *T. cruzi* infection may be influenced the milieu of the central nerves system (CNS). Although the association between leishmaniasis and PD has remained unclear, LRRK2 deficiency in DCs enhances the production of pro-inflammatory cytokines and T-cell proliferation. LRRK2 dysfunction and the initiation of inflammation due to infection might be influenced by the survival of neural cells and CNS milieu. In an in-vivo infection model, we did not investigate the effect of neurons and CNS. To prove the influence of LRRK2 dysfunction by infection, we need to examine the damage of CNS in pathogen-infected WT and LRRK2 KO mice.

In conclusion, we clarify that LRRK2 in DCs negatively regulates the production of inflammatory cytokines and antigen presentation to CD4^+^ T-cells. Our present results suggest that LRRK2 may play novel roles in both the innate immune system and the pathogenesis of various diseases such as intracellular pathogenic infection.

## 4. Materials and Methods

### 4.1. Animals

C57BL/6J (wild type: WT) mice were purchased from CLEA Japan, Inc. (Tokyo, Japan). LRRK2 exon 41-knockout (KO) mice, developed by Dr. Heather L. Melrose and Professor Matthew J Farrer and co-workers [41] on C57BL/6J mice, were used. All experiments were reviewed and approved (No. 13-06 (1 April 2013), 14-06 (1 April 2014), 15-05 (1 April 2015), 16-03 (1 April 2016), 17-03 (1 April 2017), and 17-23 (1 April 2017)) by the Animal Experimentation and Ethics Committee of Kitasato University. Mice were cared for and handled in accordance with the guidelines of the Animal Experimentation and Ethics Committee of Kitasato University.

### 4.2. Granulocyte/Macrophage-Colony Stimulating Factor (GM–CSF)

GM–CSF-producing plasmacytoma cell line X63-Ag8 was cultured in RPMI-1640 medium containing 10% heat-inactivated fetal bovine serum (FBS) (Thermo Fisher Scientific, Tokyo, Japan), 100 U/mL penicillin, and 100 μg/mL streptomycin. X63-Ag8 confluent culture supernatants were collected as the GM–CSF source.

### 4.3. Differentiation of Bone Marrow Derived Dendritic Cells (BMDCs) 

Bone marrow cells from both WT and KO mice were obtained by bone marrow washout with RPMI-1640 medium. The cell suspensions were depleted of erythrocytes by hypotonic lysis at room temperature. Bone marrow cells (2 × 10^6^) were cultured in 10 mL of RPMI-1640 medium containing 5% heat-inactivated FBS, 100 U/mL penicillin, 100 μg/mL streptomycin, 50 μM 2-mercaptoethanol, and 10% X63-Ag8 confluent culture supernatants in 100-mm Petri dishes. Another 10 mL of medium was added to the plates at day 3. Half of the culture supernatants were collected and centrifuged. The cell pellet was re-suspended in 10 mL of fresh medium, and returned to the original plate on days 5, 7, and 10. Bone marrow-derived dendritic cells (BMDCs) were collected on days 10 and 12.

### 4.4. Immunofluorescence Staining of BMDCs

BMDCs were counted using a CYTORECON CYT-1000 (GE Healthcare, Tokyo, Japan) and stained for flow cytometric analysis using combinations of the fluorochrome-conjugated monoclonal antibodies CD11b (phycoerythrin: PE) and CD11c (Pacific blue) (BioLegend, San Diego, CA, USA). The stained cells were analyzed using a MACSQuant flow cytometer (Miltenyi Biotec GmbH, Bergisch Gladbach, Germany).

### 4.5. ELISA Assay 

For detection of tumor necrosis factor-α (TNF-α) and interleukin-6 (IL-6), BMDCs from both WT and KO mice were stimulated with 500 ng/mL lipopolysaccharide (LPS) (Sigma-Aldrich, St. Louis, MO, USA) for 24 h. TNF-α and IL-6 in culture supernatants were measured using the LEGEND MAX^TM^ Mouse TNF-α ELISA kit and LEGEND MAX^TM^ Mouse IL-6 ELISA kit (Biolegend).

### 4.6. Detection of Intracellular Phosphorylated Transcription Factors

BMDCs were stimulated with 500 ng/mL LPS for 15, 30, 60, and 120 min. After incubation, LPS-stimulated DCs were treated with Phosflow Lyse/Fix buffer (Becton Dickinson, San Jose, CA, USA) warmed to 37 °C. They were then treated with Phosflow Perm buffer III (Becton Dickinson) cooled to −20 °C and stained with anti-Phospho-Erk1/2 (pT202/pY204) antibody (Alexa 488) (Becton Dickinson), anti-Phospho-p38 (pT180/pY182) antibody (PE/Cy5) (Becton Dickinson, Tokyo, Japan), and anti-Phospho-NF-κB (Ser536) antibody (Alexa 647) (Cell Signaling Technology, Tokyo, Japan). Phosphorylated-Erk1/2 (pT202/pY204), -p38 (pT180/pY182), and -NF-κB (Ser536) in LPS-stimulated BMDCs were analyzed using a MACSQuant flow cytometer (Miltenyi Biotec GmbH, Bergisch Gladbach, Germany).

### 4.7. Analysis of Antigen Presentation Ability

For analysis of antigen presentation ability, WT and KO mice were immunized by footpad inoculation with keyhole limpet hemocyanin (KLH; FujiFilm Wako, Osaka, Japan) mixed with Freund’s complete adjuvant (Nacalai Tesque, Kyoto, Japan). At 7–10 days after inoculation, the immunized mice were euthanatized under anesthesia with Isoflurane (FujiFilm Wako). Whole lymphocytes were obtained by disruption of popliteal and inguinal lymph nodes. The cell suspensions were depleted of erythrocytes by hypotonic lysis at room temperature. CD4^+^ T-cells were isolated using a CD4^+^ T-cell isolation kit II (Miltenyi Biotec) in accordance with the supplied manual, using auto-MACS (Miltenyi Biotec). CD4^+^ T-cells were stained with 5(6)-carboxyfluorescein diacetate succinimidyl ester (CFSE) (Sigma-Aldrich) for the detection of cell proliferation. BMDCs from WT and KO mice were stimulated with KLH for 16 h. After incubation, the KLH-stimulated DCs (5 × 10^3^) were co-cultured with KLH-primed CD4^+^ T-cells (5 × 10^5^) in a 96-well U-bottom plate. After 4 days of incubation, CD4^+^ T-cells were collected and stained with anti-CD4 antibody (PE) (Biolegend) and 7-amino-actinomycin D (7AAD) (Becton Dickinson). The stained cells were analyzed using a MACSQuant flow cytometer (Miltenyi Biotec).

### 4.8. Leishmania Infection In Vitro and In Vivo

For *Leishmania* infection in vitro, BMDCs (1 × 10^5^) were co-cultured with the metacyclic-promastigote form of *Leishmania* (*Leishmania*) *major* (*L.* (*L.*) *major*) at a multiplicity of infection of 1:1. After 3 days of incubation, the concentrations of TNF-α and IL-6 in the culture supernatant were measured using ELISA. For in vivo infection, the metacyclic-promastigote form of *L.* (*L.*) *major* (1 × 10^6^) was inoculated into the footpads of WT and KO mice. After inoculation, footpad thicknesses were measured periodically using micrometer calipers. At 4 days after inoculation, serum samples were collected from the tail vein under anesthesia. The concentrations of cytokines in serum samples were measured using the LEGENDplex^TM^ Mouse Th cytokine panel (Biolegend) and a MACSQuant flow cytometer (Miltenyi Biotec).

### 4.9. Statistics

Statistical analysis were performed with GraphPad Prism 8.2 (GraphPad Software Inc., San Diego, CA, USA). Statistical significance was analyzed using one-way ANOVA with Bonferroni post hoc test, two-way ANOVA with Bonferroni post hoc test, and Student’s *t* test. Differences were considered statistically significant at *p* < 0.05.

## Supplementary Materials

Supplementary materials can be found at https://www.mdpi.com/1422-0067/21/5/1890/s1.
