# Supplementary material for: Leucine-Rich Repeat Kinase 2 Controls Inflammatory Cytokines Production through NF-κB Phosphorylation and Antigen Presentation in Bone Marrow-Derived Dendritic Cells
Source: Int J Mol Sci. 2020 Mar 10;21(5):1890. doi: 10.3390/ijms21051890 (PMC7084871; doi:10.3390/ijms21051890)
Supplement: Supplementary file 1 [file ijms-21-01890-s001.pdf]

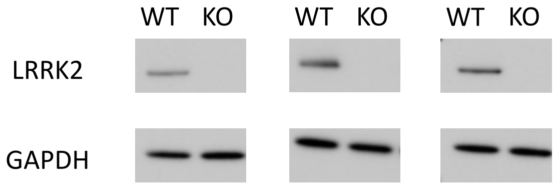

Figure S1. LRRK2 expression in BMDCs from WT and KO mice. LRRK2 expression in BMDCs was analyzed by Western blotting using primary antibodies against LRRK2 (MJFF-2) and GAPDH. Each protein was detected using horseradish-conjugated donkey anti-rabbit IgG polyclonal antibody. Data are representative of three independent experiments each involving six mice (WT n = 3, KO n = 3).

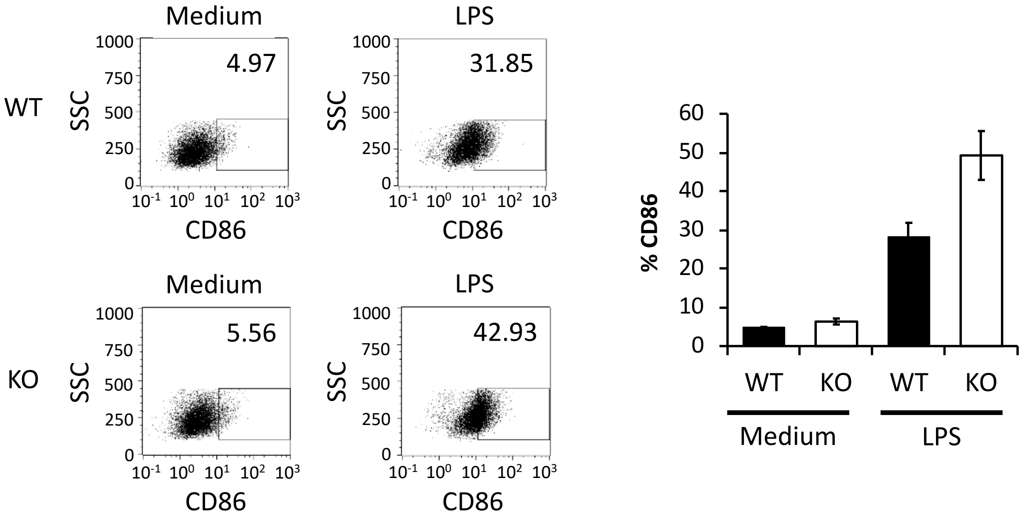

Figure S2. CD86 expression in BMDCs from WT and KO mice. BMDCs induced from WT and KO mice were stimulated with LPS. After 24 hours, the BMDCs were collected and their CD86 expression was analyzed by flow cytometry. (A) CD86 expression in unstimulated WT BMDCs. (B) CD86 expression in LPS-stimulated WT BMDCs. (C) CD86 expression in unstimulated KO BMDCs (D) CD86 expression in LPS-stimulated KO BMDCs. (E) Levels of CD86 expression in WT (black bar) and KO (white bar) mice. Data are representative of two independent experiments each involving two mice.
